# Rationale for the Cytogenomics of Cardiovascular Malformations Consortium: A Phenotype Intensive Registry Based Approach

**DOI:** 10.3390/jcdd2020076

**Published:** 2015-04-29

**Authors:** Robert B. Hinton, Kim L. McBride, Steven B. Bleyl, Neil E. Bowles, William L. Border, Vidu Garg, Teresa A. Smolarek, Seema R. Lalani, Stephanie M. Ware

**Affiliations:** 1 Divisions of Cardiology and Genetics, Cincinnati Children’s Hospital Medical Center, Cincinnati, OH 45229, USA; E-Mails: bingrbh@icloud.com (R.B.H.); teresa.smolarek@cchmc.org (T.A.S.); 2 Center for Cardiovascular and Pulmonary Research and Heart Center, Nationwide Children’s Hospital and Department of Pediatrics, Ohio State University, Columbus, OH 43205, USA; E-Mails: kim.mcbride@nationwidechildrens.org (K.L.M.); vidu.garg@nationwidechildrens.org (V.G.); 3 Division of Pediatric Cardiology, University of Utah School of Medicine, Salt Lake City, UT 84132, USA; E-Mails: steven.bleyl@utah.edu (S.B.B.); neil.bowles@hsc.utah.edu (N.E.B.); 4 Division of Cardiology, Children’s Healthcare of Atlanta, Atlanta, GA 30322, USA; E-Mail: borderw@kidsheart.com; 5 Department of Molecular and Human Genetics, Baylor College of Medicine, Houston, TX 77030, USA; E-Mail: seemal@bcm.edu; 6 Departments of Pediatrics and Medical and Molecular Genetics, Indiana University School of Medicine, Indianapolis, IN 46202, USA

**Keywords:** genetics, genomics, pediatrics, cardiovascular malformation, registry, chromosome microarray, copy number variation

## Abstract

Cardiovascular malformations (CVMs) are the most common birth defect, occurring in 1%–5% of all live births. Although the genetic contribution to CVMs is well recognized, the genetic causes of human CVMs are identified infrequently. In addition, a failure of systematic deep phenotyping of CVMs, resulting from the complexity and heterogeneity of malformations, has obscured genotype-phenotype correlations and contributed to a lack of understanding of disease mechanisms. To address these knowledge gaps, we have developed the Cytogenomics of Cardiovascular Malformations (CCVM) Consortium, a multi-site alliance of geneticists and cardiologists, contributing to a database registry of submicroscopic genetic copy number variants (CNVs) based on clinical chromosome microarray testing in individuals with CVMs using detailed classification schemes. Cardiac classification is performed using a modification to the National Birth Defects Prevention Study approach, and non-cardiac diagnoses are captured through ICD-9 and ICD-10 codes. By combining a comprehensive approach to clinically relevant genetic analyses with precise phenotyping, the Consortium goal is to identify novel genomic regions that cause or increase susceptibility to CVMs and to correlate the findings with clinical phenotype. This registry will provide critical insights into genetic architecture, facilitate genotype-phenotype correlations, and provide a valuable resource for the medical community.

## 1. Introduction

Chromosomal abnormalities account for 12%–14% of all live born cases and 20%–33% of fetal cases of congenital cardiovascular malformations (CVMs), indicating that the proper genetic control of cardiac development is essential for normal anatomic structure [1,2,3,4]. The complex development of the heart suggests the involvement of numerous genes in normal cardiac morphogenesis and, hence, numerous chromosomal loci [5]. In addition, syndromic CVMs can be quite difficult to distinguish from isolated CVMs, especially in early childhood [6,7]. Since early diagnosis of any genetic condition can inform clinicians in how best to optimize medical management, developmental intervention, and therapy, it becomes even more critical to more precisely delineate etiology. Additional benefits of a precise diagnosis include determination of prognosis and more accurate counseling about recurrence risk [8].

CVMs in humans commonly display incomplete penetrance and variable expressivity, with extensive allelic heterogeneity. This complex inheritance suggests a central contribution of as yet unidentified genetic modifiers. The high frequency of CVMs and their evident heterogeneity poses substantial problems in understanding the genetic causes underlying these common birth defects. However, by grouping CVMs that are mechanistically related, such as left ventricular outflow tract obstructive defects (LVOTO), heritability patterns can be identified [9,10,11,12,13,14]. These analyses have demonstrated that mild CVMs such as bicuspid aortic valve (BAV) may represent a *forme fruste* of severe CVMs, e.g., hypoplastic left heart syndrome (HLHS). Genetically mediated cardiac diseases that may not be present or ascertained at birth, e.g., BAV (present in ~2% of the population) or aortic dilation, are much more common than previously thought. When considering the etiology of CVMs, as opposed to the proportion of CVM cases that manifest as disease at birth, the incidence increases to approximately 5%.

Increasingly sophisticated genetic analyses, including chromosome microarray analysis (CMA) and next generation sequencing, are improving the ability to identify genetic causation and susceptibility of CVMs. CMA can also be used to analyze regions of homozygosity, which may unmask an autosomal recessive disorder. In addition, abnormalities in the parent of origin or imprinted chromosomal material (e.g., uniparental disomy) can contribute to CVMs and are detectable by current tests for copy number abnormalities [15,16]. CMA is standard of care testing for patients with developmental disability or multiple congenital anomalies and is considered first line testing for many patients with CVMs [17]. Copy number variation (CNV) refers to genetic deletions or duplications that are not identifiable by traditional chromosome analysis. These alterations may result from recombination within the genome, typically as a result of instability of regional genetic architecture, and vary in size. CNVs are more common than single nucleotide variants, and therefore generate important variation in the genome. CNVs are common, comprising approximately 12% of the genome of an individual and typically have no phenotypic consequence. However, CNVs containing dosage sensitive gene(s) result in phenotypic abnormalities due to alterations in gene expression, suggesting CNVs may represent major genetic modifiers in addition to their role in disease causation. The mechanisms of CNVs in disease have been the subject of recent reviews [18,19,20,21].

CNVs containing dosage sensitive genes are a well-described cause of genomic disorders associated with CVMs such as 22q11.2 deletion syndrome and Williams-Beuren syndrome, where *TBX1* and *ELN*, respectively, are critical dosage sensitive genes involved in the pathogenesis of these syndromes. These recurrent CNVs are associated with highly penetrant CVMs when haploinsufficient. More recent studies support a role for rare recurrent CNVs in CVMs with extracardiac malformations, as well as isolated CVMs [22,23]. It is estimated that CNVs contribute to 3%–25% of CVMs associated with extracardiac abnormalities and 3%–10% of isolated CVMs reviewed in [24,25] and this may be a significant underestimate given the current lack of understanding surrounding the contribution of genetic modifiers to the genetic basis of CVM.

Classification approaches for CVMs continue to present significant challenges for both clinicians and researchers. *The Atlas of Congenital Cardiac Disease*, based on Maude Abbott’s systematic catalogue of CVM gross pathology published in 1908 is the first classification system for clinical pediatric cardiology [26]. Subsequently, beginning in earnest in the 1950s, the rapid evolution of surgical intervention for CVMs defined a detailed clinical taxonomy that was based on anatomy and physiology [27]. Organizing malformations requires consideration of cause, and only recently, in the context of significant strides in our understanding of the genetic basis underlying CVMs, have there been efforts to reconcile etiologic factors in a clinically meaningful manner and integrate this information into the existing classification scheme [8,28]. As our understanding of cardiac morphogenesis improved, hypothetical classification paradigms were developed that took into account the developmental relationships of lesions (in addition to the anatomic relationships previously described) to increase confidence in grouping [29]. As the genetic basis of CVMs has emerged, it has become clear that this knowledge will challenge our clinical taxonomy in fundamental ways. The National Birth Defects Prevention Study (NBDPS) developed an exhaustive taxonomy that organizes CVMs in multiple ways, including the most specific definition of a single defect (the “splitting” approach) and the broadest groupings (the “clumping” approach), as well as intermediate levels that allow flexibility with the analysis of common associations [30]. A classification system that incorporates etiologic factors as well as careful clinical phenotyping is necessary for clinical and research advances alike, including the large scale multi-site efforts required to address the relatively small frequency of CVMs per center.

A goal of the registry is to collect and annotate cytogenetic and phenotypic findings in patients with CVMs in a comprehensive and standardized manner to promote an understanding of the genetic basis of cardiac disease. A lack of systematic deep phenotyping of CVMs, resulting from the complexity and heterogeneity of malformations, has obscured genotype-phenotype correlations and contributed to a lack of understanding of disease mechanisms. Defining critical genes within CNVs, establishing rare recurrent CNVs associated with CVMs, and defining cardiac phenotypes of genomic disorders are all important objectives of this Registry. The high frequency of CVMs, large number of genes playing a role in normal cardiac development, and incomplete penetrance of phenotypes suggests that large numbers of cases will be required to identify causes and determine associations and susceptibility factors. Given the established phenotypic variability associated with genetic causes of CVMs, it is necessary to use a detailed and etiology-centric classification system to leverage the information learned from these associations. The CCVM Registry is a cost-effective resource to identify novel loci associated with isolated or syndromic CVMs, establish genotype-phenotype correlations, and facilitate discovery of new candidate genes for normal and abnormal cardiac development.

## 2. Experimental Section

### 2.1. Registry Organization

The CCVM Consortium is a collaborative group of 6 pediatric clinical centers, with different areas of expertise but common interests and broad experience in the genetics of CVMs, cytogenetics, pediatric cardiology, including specifically echocardiography, clinical genetics, and database/registry/biobank expertise.

The Investigator Group encompasses clinical expertise in cardiology (Border, Garg, Hinton, with specialized imaging expertise by Border, Hinton), clinical genetics (Bleyl, Lalani, McBride, Ware), and cytogenetics (Lalani, Smolarek,); research expertise in human genetics (Bleyl, Bowles, Garg, Hinton, Lalani, McBride, Ware), cardiac development (Bleyl, Garg, Hinton, Ware), clinical research (Border, McBride); and database/registry/biobank experience (Bleyl, Bowles, Hinton, McBride, Ware).

### 2.2. Study Population and Study Design

This is a cohort study of abnormal CMA cases in patients for whom echocardiography has been performed. To be eligible for the study, a patient is required to have had an abnormal CMA result, defined as a result interpreted to be clinically significant, disease causing, or of unknown clinical significance. Patients with no CMA results or normal testing are excluded. Of note, an iterative search process is implemented such that patients with abnormal CMA in whom no echocardiogram has been performed will remain potentially eligible, thus capturing patients with later diagnoses. Patients with isolated abnormalities in cardiac rhythm will be excluded from this study. The Institutional Review Boards of all participating study sites have approved the Registry and data use agreements have been executed to allow sharing of limited datasets.

### 2.3. Cytogenetic Data Elements

As certified clinical diagnostic laboratories, cytogenetics laboratories at the 6 sites have standardized requirements for processing and tracking patient samples as well as analyzing, interpreting, and recording analyses. Cytogenetic variables from each site have been standardized for collection (Table 1). Each patient sample is assigned a case number with a unique identifier, and the cytogenetic laboratory maintains a large database of case numbers, results, and clinical interpretation. A DNA repository of samples is maintained long term for auditing and regulatory compliance. Due to the high standards for regulatory compliance and clinical interpretation inherent in the operation of a clinical diagnostic laboratory, the analyses represent a very well validated dataset. CMA frequently uncovers novel submicroscopic abnormalities [23]. In some cases, results are confirmed by a second method, most often fluorescence in situ hybridization. In addition, in cases of uncertain significance, parental samples are obtained when available to determine whether the submicroscopic gain or loss is *de novo*, to aid interpretation of pathogenicity. Literature and database searches are also performed for better interpretation of the data. CMA analytic tools are routinely used for interpretation of novel variants. These interpretation results are maintained in the Registry. Table 1 shows variables collected with example results shown for 22q11.2 deletion syndrome.

**Table 1:** Cytogenetic Data Elements.

Variable	Example
Platform	SNP
Chromosome	22
Cytolocation	22q11.21
Position Beginning	17,074,487
Position End	19,806,645
Version	HG18
CNV Value	1
Gain/Loss	Loss
Gene	USP18, DGCR6, PRODH, DGCR2, DGCR14, TSSK2, GSC2, SLC25A1, CLTCL1, HIRA, MRPL40, UFD1L, CDC45L, CLDN5, SEPT5, GP1BB, TBX1, GNB1L, TXNRD2, COMT, ARVCF, DGCR8, TRMT2A, RANBP1, ZDHHC8, RTN4R, DGCR6L, GGTLC3, RIMBP3, ZNF74, SCARF2, MED15, PI4KA, SERPIND1, SNAP29, CRKL, LZTR1, THAP7, P2RX6, SLC7A4, BCRL2
Inheritance	*De novo*
Size	2732158 bp
Probes	N/A
Number of Probes	1,140,419
Analysis	arr 22q11.21(17,074,487 − 19,806,645) × 1
Result	Consistent with a single copy loss of genomic segment or heterozygous deletion of approximately 2.73 Mb on chromosome 22q11.21. This finding confirms the concurrent FISH analysis, which showed a 22q11.2 deletion associated with DiGeorge syndrome. The deleted 22q11.21 region overlaps with the region associated with the 22q11.2 deletion syndrome (DiGeorge syndrome) and contains several disease genes including the TBX1 gene known to cause clinical phenotypes seen in patients with the syndrome. The deleted region is proximal to the SMARCB1 locus and no deletion of the SMARCB1 gene was detected in this assay.

### 2.4. Detailed Cardiac and Extra-Cardiac Phenotyping

Comprehensive segmental anatomy is collected from echocardiograms performed for clinical care at each site. All primary echocardiogram data is evaluated directly at each site and classified to ensure exceptionally accurate phenotyping, which is an absolutely essential prerequisite for meaningful analysis and interpretation of genetic data. Non-cardiac medical diagnoses (e.g., dysmorphic features, developmental delay, neurologic findings) are recorded in the composite database using ICD-9 and now ICD-10 coding. Criteria established by the National Birth Defects Prevention Study (NBDPS) are being used to collect extensive phenotype information regarding the CVMs [30]. These criteria have been adapted to optimize capture of all cardiac defects that may be attributed to genetic causes, using the term “malformation” broadly with the assumption that the vast majority of cases of pediatric heart disease has a genetic basis (Table 2). Adaptations include the addition of other forms of pediatric heart disease, including rare developmental aberrations that were not included in the NBDPS scheme, such as arteriopathy, coronary artery abnormalities, and latent cardiovascular disease that is not present at birth but has been associated with genomic abnormalities, such as cardiomyopathy and aortopathy, all lesions that might now be considered ‘malformations’ in a genetic and pathogenesis sense if not strictly pathologic. Importantly, functional deficits of unclear significance are captured in addition to structural defects, e.g., left ventricular systolic dysfunction. In addition, subclinical CVMs are included, such as BAV. In light of added items, new associations are added. Finally, persistent fetal communications that may represent malformation are tracked when present beyond the first year of life, e.g., patent ductus arteriosus. In addition, there is a free text field allowing capture of additional items that are not systematically screened for but may be considered subclinical and abnormal, for example isolated left superior vena cava. As specified by the original NBDPS, each case was assigned a classification based on three axes: cardiac phenotype, cardiac complexity, and the presence and type of extracardiac malformations [30]. The CCVM modified classification scheme is shown in the case report form in Supplemental Table S1.

**Table 2:** Basic Tenets of National Birth Defect Prevention Study (NBDPS) Classification and Cytogenomics of Cardiovasscular Malformations (CCVM) Consortium Modifications.

The NBDPS describes each patient’s cardiovascular malformation (CVM) as a specific lesion and groups the lesion in fine, intermediate and coarse levels of detail to increase precision and allow flexibility in analyses [28]
The NBDPS describes complexity of CVM and patterns of associated anomalies
The CCVM Registry includes latent types of pediatric heart disease that have an established genetic etiology, including cardiomyopathy and aortopathy
The CCVM Registry includes vasculopathies and coronary artery abnormalities
The CCVM Registry includes subclinical CVMs, such as bicuspid aortic valve
The CCVM Registry includes persistent fetal connections when present beyond one year of life, such as patent ductus arteriosus
The CCVM Registry includes functional deficits that may identify emerging defects, such as left ventricular systolic dysfunction

All echocardiographic reports are reviewed at a single center (IU) and CVM phenotype designation is made at that time. Due to differences in reporting across study sites, regular conference calls address designation choices and studies of uncertain classification. A cardiologist will over-read 10% of studies, excluding straightforward lesions (e.g., isolated septal defects), and the central study site will address discrepancies.

### 2.5. Data and Information Management

Using Good Clinical Data Management principles and processes, as well as institutionally sanctioned standard operating procedures, the DCC ensures that data are valid, reliable and accurate leading to reproducible results. Demographic and cardiac indications/echocardiography results are recorded onto paper case report forms (CRFs) and sent to IU DCC for entry into a REDCap (Research Electronic Data Capture) database. Cytogenetic data are de-identified and transferred securely. The DCC is responsible for maintaining the existing database system, importing external data, programming data quality checks, query resolution, generation of reports, data exports and preparation for analysis. A detailed data management plan is be maintained to document all processes. A data use agreement outlines the proper use of the data and publication plans.

The study complies with all Federal and local privacy laws and regulations, including the Department of Health and Human Services (HHS) Protection of Human Subjects Regulations (45 CFR part 46 and 21 CFR parts 50) and The Health Insurance Portability and Accountability Act (HIPAA) Privacy Rule. Phenotypic information is linked to the cytogenetic information and a unique identifier number will be assigned within the composite database. REDCap provides a secure, web-based application that is flexible and provides (1) an intuitive interface for data entry and real time validation rules at the time of entry; (2) HIPAA-compliant and 21 CFR Part 11-ready audit trails for tracking page views, data manipulation and export procedures; (3) record locking and electronic signature functions; (4) fine grained control of user rights to view/manipulate data; (5) a report builder for reporting, monitoring and querying patient records; and (6) automated export procedures for seamless data downloads [31].

## 3. Results and Discussion

### 3.1. Study Population Enrollment

The cohort is geographically, racially, and ethnically diverse which represents a strength for genetic studies. Current and projected enrollment is shown in Table 3. The Utah site is predicted to have fewer cases, but a higher proportion will be linked in large non-syndromic CVM families, due to the availability of extended family data from the Utah Population Database, a computerized data warehouse with an extensive set of genealogy data (~12 million records) linked to medical information.

**Table 3:** Current and Projected Enrollment.

Site	Current Cases	Yr1	Yr2	Yr3	
BCM	305	75	85	85	
CCHMC	174	55	60	60	
Nationwide	76	55	60	60	
Emory/CHOA	67	30	35	40	
Utah	N/A	15	20	20	
Totals	622	230	260	265	1377

### 3.2. Genetic Descriptors

As part of the Registry, we expect to capture known genetic syndromic diagnoses that are commonly associated with CVMs such as 22q11.2 deletion syndrome and its reciprocal duplication, 22q11.2 duplication syndrome, and Williams-Beuren syndrome and its reciprocal duplication, 7q11.23 duplication syndrome. In addition, trisomy 21 may be detected by CMA in infants in whom a diagnosis of Down syndrome was not suspected and CMA was performed. In order to determine the degree to which these common diagnoses made by CMA contribute to the registry, we performed manual review of the initial 291 records. The results are shown in Table 4. These data demonstrate that these well-described genetic syndromes do not comprise a majority of results for subjects with CVMs and abnormal CMA findings within the Registry. Epidemiologic data from the Baltimore Washington Infant Study indicated that approximately 76% of cases of CVMs were isolated, 12% resulted from aneuploidies such as trisomy 13, 18, 21, and Turner syndrome, and 12% encompassed Mendelian disorders, clinical syndromes, genomic disorders, or other complex phenotypes suggestive of genetic syndromic conditions. Because we anticipate that the majority of patients with aneuploidies will not be diagnosed using CMA, the prevalence of the most common genetic syndromic conditions associated with CVMs in the Registry appears to approximate epidemiologic estimates.

### 3.3. Cardiac Phenotyping

**Figure 1:**
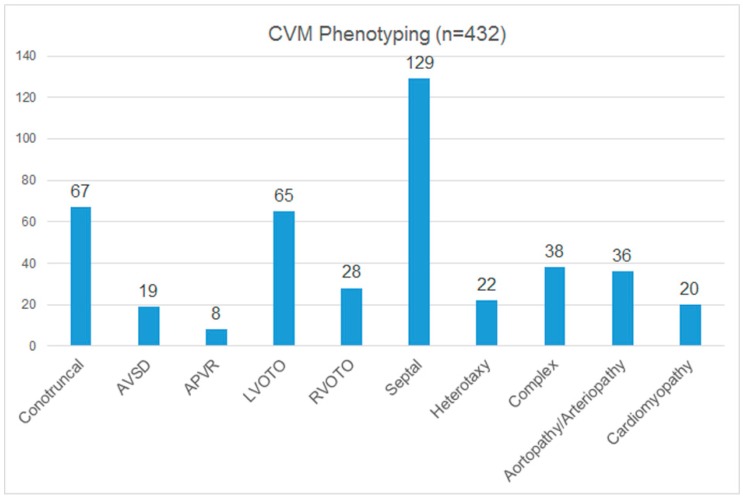
Cardiac classification groups for CCVM registry. AVSD (atrioventricular septal defects); APVR (anomalous pulmonary venous return); LVOTO (left ventricular outflow tract obstruction); RVOTO (right ventricular outflow tract obstruction).

A subset of the current cases identified as having CMA abnormalities have undergone cardiac classification. The results are shown in Figure 1, which shows the highest level subgrouping of CVMs. As expected, septal defects are the most common. The conotruncal and LVOTO categories are the next most common groups of defects, with approximately equivalent numbers. Heterotaxy CVM numbers are slightly higher than might be expected, perhaps reflecting the high prevalence of heterotaxy in Texas and the overrepresentation of cases from that region in the current Registry. Importantly, aortopathy, arteriopathy, and cardiomyopathy, all defects not captured by the NBDPS, are well represented in this cohort of patients with CMA abnormalities.

**Table 4:** Prevalence of Common Genetic Syndromes with CVM in Registry Database.

Genetic Syndrome	N in Registry ^1^	% of Registry Cases
22q11.2 deletion syndrome (DiGeorge, Velocardiofacial syndrome)	19	6.5%
22q11.2 duplication syndrome	3	1.0%
Williams-Beuren syndrome (7q11.23 deletion)	12	4.1%
7q11.23 duplication syndrome	3	1.0%
Trisomy 21	1	0.3%
Total	38	13.0%

^1^ Based on review of 291 cases.

As the clinical genetic testing paradigms for CVMs evolve, we expect to capture increasing numbers of isolated CVM subjects. It has been shown in a cohort of infants with CVMs that fewer patients were undergoing genetic testing than would be predicted to have syndromic disease based on epidemiologic data [10,32]. In addition, many patients who do undergo genetic testing have multiple, redundant tests resulting in cost inefficiencies [33,34]. While a conventional tiered approach to genetic testing is important, these studies have identified circumstances when broader testing may be indicated. Four of the six sites involved with the Registry have implemented institution-specific approach to infants with cyanotic CVMs, with standardized approaches for genetic testing and/or cardiovascular genetics consultation, increasing the comprehensive evaluation of infants with CVMs. Recent studies showing the diagnostic yield of CMA in isolated CVM cases provides rationale for clinical testing [24,35,36]. The CCVM Registry may provide a repository to determine the yield of testing within certain classes of CVMs, thereby providing an improved ability to implement genetic testing appropriately.

### 3.4. Proof of Principle: Anticipated Outcomes

#### 3.4.1. Delineation of Cardiac Features for Well Characterized Genomic Disorders

Because CVMs are the most common birth defects, many well-characterized genomic disorders have CVMs associated as part of the phenotype. In many cases, careful description of the CVMs associated with these disorders has been lacking, and for some genetic syndromes, no specific information exists about the type of heart defects identified. An anticipated and important function of the CCVM Registry will be to provide deep phenotyping of CVMs for a number of recurrent genomic disorders that have not been carefully studied. For example, we recently published a case series of eight patients with 7q11.23 duplication and aortopathy [37]. The 7q11.23 microduplication syndrome is a genomic disorder with an emerging clinical phenotype including dysmorphic features, hypotonia, developmental delay with prominent speech delay, and autistic features. CVMs, most commonly patent ductus arteriosus, have been reported in a subset of cases. We identified a series of eight pediatric patients and one adult with 7q11.23 microduplication syndrome, all of whom had aortic dilation, the opposite vascular phenotype of the typical supravalvar aortic stenosis found in Williams-Beuren syndrome. The ascending aorta was most commonly involved, while dilation was less frequently identified at the aortic root and sinotubular junction. Inclusion and systematic evaluation of aorta dimensions, as part of the comprehensive phenotyping approach, allowed the identification of these patients. These initial patients were ascertained at a single institution, and CCVM Registry participation allowed the identification of additional subjects. The incidence of 7q11.23 duplication syndrome is estimated at 1/13,000 to 1/20,000 births and therefore no single institution would be expected to have a large cohort of patients. This example indicates that the CCVM Registry is an important resource for better defining cardiac features in genetic syndromes that are still being characterized or for which cardiac features are less common. Importantly, in this example the findings led to a management recommendation for cardiovascular surveillance in patients with 7q11.23 microduplication syndrome.

#### 3.4.2. Identification of Novel Loci that Confer CVM Susceptibility

The widespread use of CMA for diagnosis in patients with developmental disability or congenital anomalies has led to the identification of many new genomic disorders as well as CNVs of uncertain clinical significance. There are a large number of genes predicted to impact cardiac development and therefore we have hypothesized that some submicroscopic deletions or duplications associated with CVMs may be quite rare. Rare, recurrent CNVs associated with CVMs are important to identify because they allow further delineation of the clinical spectrum of disease, allow refinement of the critical region for specific phenotypic features, and provide a mechanism to identify novel candidate genes for CVMs. Recently, Lalani *et al*., undertook a large study of rare, recurrent CNVs associated with CVMs in patients with abnormal CMA findings and extracardiac anomalies [22] and identified 16 unique CNVs, 12 of which were novel loci, including 16q24.3 loss and 2q31.3–q32.1 loss. In addition, the study narrowed critical intervals in three well-recognized genomic disorders with CVMs.

#### 3.4.3. Identification of Novel Genes Causing CVMs

Coarctation of the aorta (CoA) and HLHS, both LVOTO defects, have been reported in rare individuals with large terminal deletions of chromosome 15q26. However, no single gene important for left ventricular outflow tract development has been identified in this region. Clinical CMA testing through the Kleberg Laboratory at Baylor College of Medicine identified two half-siblings with CoA with a 2.2 Mb deletion on 15q26.2, inherited from their mother, who was mosaic for this deletion. This interval contains a single gene, *MCTP2* (multiple C2-domains with two transmembrane regions 2). The CCVM Registry can be used to further investigate candidate genes identified in novel CNVs and/or to further narrow critical regions for loci previously associated with CVMs. These candidate genes can be further investigated using genetic and developmental approaches. In the case of *MCTP2*, gene-specific array screening in 146 individuals with non-syndromic LVOTO defects identified another individual with a *de novo* 41 kb intragenic duplication within *MCTP2*, predicted to result in premature truncation, p.F697X [38]. Alteration of *Mctp2* gene expression in *Xenopus laevis* embryos by morpholino knockdown and mRNA overexpresssion resulted in failure of proper outflow tract development, confirming the functional importance of this dosage sensitive gene for cardiogenesis and illustrating the importance of precise phenotyping. Our results identify *MCTP2* as a novel genetic cause of CoA and related cardiac malformations.

In addition, we identified a patient with unbalanced atrioventricular septal defect (AVSD) and hypoplastic left ventricle who harbored an ~0.3 Mb deletion on chromosome 3p14.1 [39]. The deletion encompassed the first four exons of *FOXP1*, a gene critical for normal heart development that represses cardiomyocyte proliferation and expression of Nkx2.5. Identification of this candidate gene led to sequence based approaches within patients with CVMs to determine whether single nucleotide variants in *FOXP1* cause or increase susceptibility to CVMs. It should be noted that the CCVM Registry only captures patients with abnormal CMA, and thus no sequence-based interrogation has been performed. Sequencing *FOXP1* in 82 patients with AVSD or HLHS led to the identification of two patients with a variant that showed abnormal transactivation in functional assays as well as alterations in cardiomyoblast proliferation, suggesting that haploinsufficiency of *FOXP1* is associated with human CVMs.

### 3.5. Approaches for Genetic Identification of CVMs

There is a clear need presently for combining clinical genetic testing with deep phenotyping, and this need requires flexibility on both the genotype and phenotype spectrums due to substantial heterogeneity. In addition to careful cardiac phenotyping, there is a growing appreciation that extra-cardiac organ systems should be considered in all cases of CVMs, and there is a growing appreciation of central nervous system abnormalities that significantly impact both a patient’s medical status and his or her quality of life. A strength of this Registry is the careful cardiac phenotyping combined with the systematic collection of extracardiac findings, bolstering analyses focusing on the genetic underpinnings of these specific findings and associations. For example, we recently reported twins with progressive aortopathy, recurrent dissections and an *ACTA2* mutation [40]. Interestingly, in addition to illustrating the potential for genotype-phenotype correlations that directly impact clinical care, this example also illustrates the potential use of non-cardiac findings to direct cardiac care. These twins were diagnosed with mydriasis years prior to their aortopathy being recognized, and because mydriasis is a manifestation of smooth muscle dysfunction this might prompt a heightened index of suspicion for aortopathy and therefore surveillance. These types of associations challenge previous system based models of disease and allow paradigms whereby genetic information is integrated into clinical practice. A limitation of the Registry is that it is currently focused only on CNVs as a mechanism for CVM causation and susceptibility. In its present form, it does not incorporate sequence based approaches to genetic diagnosis, and this is a clear need for future iterations.

Patients with CVMs are managed clinically by pediatric cardiologists and pediatric cardiothoracic surgeons primarily, but several types of specialists are now routinely involved in the delivery of comprehensive care, importantly including increasing numbers of health care providers with specific expertise in cardiovascular genetics such as geneticists and genetic counselors [41,42]. Distinct nomenclature systems have been developed in both disciplines. For example, surgical paradigms of CVM have been systematically archived by the Society of Thoracic Surgeons (STS) by lesion [43] using an extreme reductionist approach to classification. The Pediatric Heart Network is a multi-site network that conducts clinical trials, and uses project specific phenotyping, but is charged with partnering with the Pediatric Cardiovascular Genomic Consortium, a rapidly growing registry of CVM patients and biorepository of DNA that uses a variation of the Fyler classification scheme based on anatomy and physiology [44,45]. The CCVM Registry presented in this manuscript uses an inclusive approach to an etiology centric classification scheme. When considering cause, it is important to group lesions appropriately and avoid misclassification that may confound interpretation. Importantly, these approaches to phenotypic details are different, potentially confounding analyses. A central need in the field is for consensus regarding careful phenotyping, and this will be made more challenging by the emerging molecular taxonomy. Because the genetic basis of CVMs, and more broadly pediatric heart disease, is characterized by both phenotypic and genetic heterogeneity, with an anticipated central role for common major modifiers, a defined genetic understanding of CVMs will greatly inform complete phenotyping, potentially impacting diagnosis, prognosis and treatment decisions, as well as counseling for families. Therefore, it is crucial that the larger CVM community reconcile different approaches to careful phenotyping to fully leverage the potential of current large registries.

Molecular taxonomies, defined by human genetics, are going to impact established clinical taxonomies and change approaches to care, resulting in the increased use of preventive and proactive strategies. The definition of a syndrome is less straightforward than in previous years, as single gene defects may result in apparently isolated CVMs in some individuals, and CVMs with extracardiac findings in others. Therefore, one specific opportunity lies in the ability to delineate both genetic modifiers and phenotypic variability, e.g., the same gene mutation resulting in varying CVMs in a given family or within a known syndrome [46,47]. Given the analytic challenges involved, recent recommendations from the American Heart Association state that Centers with Cardiovascular Genetics Centers should direct testing and interpretation and participate in subsequent management decisions, especially as exome testing becomes more common [48]. The most effective approaches for delivering cardiovascular genetics services have not been completely defined, and the composition of clinics and expertise can vary [49]. Coordinated and uniform practices in cardiovascular genetics will be increasingly important as the causes of heritable CVMs are identified, risk variants are defined, and personalized medicine efforts become mainstream [49]. To further these efforts, it is imperative that the field reconcile different ways of approaching both genetic and phenotypic schemes. The success of the Children’s Oncology Group has been attributed in large part to overcoming these very challenges. In addition, they have been successful at reconciling registries and compliance issues between study sites and using collective biospecimens [50,51] and may serve as an ideal model for pediatric heart disease. Ultimately, consensus on phenotyping between centers is necessary for national and international network, consortium and registry research efforts to fully realize their potential and truly impact patient care.

## 4. Conclusions

Identification of the genetic causes of CVMs has been challenging. Although the developmental basis for many cardiac defects has been investigated in model organisms, identification of genetic causes in human CVMs has been less successful. The current experimental framework for studying CVMs is clearly deficient. The attempt to translate bench (animal models) to bedside (identification of human mutations) has not been straightforward. This has led to the paradigm that CVMs are too heterogeneous and multifactorial in origin to genetically characterize except in rare familial cases, resulting in stagnation of clinical and translational investigation and diagnostic test development. The development of the CCVM Consortium is an important step toward achieving the goal of identifying the genetic basis of CVMs. Using innovative technology, clinical expertise, and clinical diagnostic laboratory expertise, this alliance will determine the importance of structural genome variation in CVMs and enable the identification of novel loci, delineation of genetic syndromes, and genotype-phenotype analyses. In turn, this will facilitate the development of disease specific therapy, accurate diagnostic algorithms, effective management, and assignment of precise recurrence risk.

## Supplementary Materials

Supplementary materials can be accessed at: http://www.mdpi.com/2308-3425/2/2/0076/s1.
