# Supplementary material for: Rationale for the Cytogenomics of Cardiovascular Malformations Consortium: A Phenotype Intensive Registry Based Approach
Source: J Cardiovasc Dev Dis. 2015 Apr 29;2(2):76–92. doi: 10.3390/jcdd2020076 (PMC5753096; doi:10.3390/jcdd2020076)
Supplement: Supplementary file 1 [file jcdd-02-00076-s001.pdf]

Supplementary Materials

Table S1. Case Report Form

**Demographics**	
**Date of Birth:**	|__|__|-|__|__|-|__|__|__|__| M M - D D - Y Y Y Y
**Sex:** *(select only one response)*	☐ Male<br>☐ Female<br>☐ Not Reported/Unknown
**Ethnicity:** *(select only one response)*	☐ Hispanic or Latino<br>☐ Not Hispanic or Latino<br>☐ Not Reported/Unknown<br>☐ Patient Refused
**Race:** *(select only one response)*	☐ American Indian or Alaska Native<br>☐ Asian<br>☐ Black or African American<br>☐ Native Hawaiian or Other Pacific Islander<br>☐ White<br>☐ Other, specify: ______________________<br>☐ Not Reported/Unknown
**Medical History**	
**Does the Subject have any Medical History Findings to Record?**	☐ Yes ☐ No
Only complete the CRF if data is not included in a data transfer.	

	Diagnosis	ICD9 Code
1.	______________________	___ ___ ___. ___ ___
2.	______________________	___ ___ ___. ___ ___
3.	______________________	___ ___ ___. ___ ___
4.	______________________	___ ___ ___. ___ ___
5.	______________________	___ ___ ___. ___ ___
6.	______________________	___ ___ ___. ___ ___
7.	______________________	___ ___ ___. ___ ___
8.	______________________	___ ___ ___. ___ ___
9.	______________________	___ ___ ___. ___ ___
10.	______________________	___ ___ ___. ___ ___
11.	______________________	___ ___ ___. ___ ___
12.	______________________	___ ___ ___. ___ ___
13.	______________________	___ ___ ___. ___ ___
14.	______________________	___ ___ ___. ___ ___
15.	______________________	___ ___ ___. ___ ___
16.	______________________	___ ___ ___. ___ ___
17.	______________________	___ ___ ___. ___ ___
18.	______________________	___ ___ ___. ___ ___
19.	______________________	___ ___ ___. ___ ___
20.	______________________	___ ___ ___. ___ ___
21.	______________________	___ ___ ___. ___ ___
22.	______________________	___ ___ ___. ___ ___
23.	______________________	___ ___ ___. ___ ___
24.	______________________	___ ___ ___. ___ ___

Table S1. *Cont.*

Cardiac Complexities (*Read 1*)

Date of Read: |__|__|-|__|__|-|__|__|__|__|
M M - D D - Y Y Y Y

Reader's Initials: |__|__|__| (XXX, X-X)

Echo Findings:
- ☐ Abnormal
- ☐ Normal
- ☐ Cannot Evaluate
- ☐ Grossly Normal
  - ☐ Normal study with PFO/PDA at <1yr (additional imaging not available to confirm closure)
    (*select all that apply*) ☐ PFO ☐ PDA
  - ☐ Normal based on incomplete or limited study/studies

Completed by: ☐ Cardiologist ☐ Other Personnel

Cardiac Malformations (*Read 1*)

If applicable, select only one primary diagnosis from the Cardiac Malformations section. If the relevant diagnosis is not listed, choose "Other" and specify at the bottom of the chart.

	Level Three	Level Two	Level One
☐	Conotruncal	Truncus	Truncus
☐	Conotruncal	IAA, Type B	IAA, B
☐	Conotruncal	IAA, nos	IAA, nos
☐	Conotruncal	d-TGA	d-TGA-IVS
☐	Conotruncal	d-TGA	d-TGA-IVS + LVOTO (PS)
☐	Conotruncal	d-TGA	d-TGA-IVS + RVOTO (AS, COA)
☐	Conotruncal	d-TGA	TGA nos
☐	Conotruncal	d-TGA	TGA os
☐	Conotruncal	d-TGA	d-TGA-VSD
☐	Conotruncal	d-TGA	d-TGA-VSD + LVOTO (PS)
☐	Conotruncal	d-TGA	d-TGA-VSD + RVOTO (AS, COA)
☐	Conotruncal	Fallot	TOF
☐	Conotruncal	Fallot	TOF, absent pulmonary valve
☐	Conotruncal	Fallot	PA-VSD (TOF anatomy)
☐	Conotruncal	Fallot	DORV-TOF type
☐	Conotruncal	DORV-TGA	DORV-TGA type
☐	Conotruncal	DORV-other	DORV, os
☐	Conotruncal	DORV-other	DORV, nos
☐	Conotruncal	VSD, conov	VSD conov
☐	Conotruncal	IAA, Type B | Truncus	IAA, B + truncus
☐	AVSD	AVSD	AVSD, unspecified
☐	AVSD	AVSD	ASD-1
☐	AVSD	AVSD	VSD, inlet type
☐	AVSD	AVSD	AVSD, complete
☐	AVSD	AVSD	AVSD, transitional
☐	AVSD	AVSD	AVSD + LVOTO
☐	AVSD	AVSD	AVSD + RVOTO

Table S1. *Cont.*

	Cardiac Malformations (*Read 1—continued*)		
	**Level Three**	**Level Two**	**Level One**
☐	APVR	TAPVR	TAPVR
☐	APVR	TAPVR	TAPVR + RVOTO
☐	APVR	TAPVR	TAPVR + LVOTO
☐	APVR	PAPVR	PAPVR
☐	APVR	PAPVR	PAPVR + RVOTO
☐	APVR	PAPVR	PAPVR + LVOTO
☐	APVR	Cor Triatriatum	Cor Triatriatum
☐	LVOTO	HLHS	HLHS-IVS
☐	LVOTO	HLHS	HLHS + VSD
☐	LVOTO	HLHS	HLHS + APVR
☐	LVOTO	IAA, Type A	IAA, A
☐	LVOTO	Coarctation	COA-IVS
☐	LVOTO	AS	AS
☐	LVOTO	AS | Coarctation	AS + COA
☐	LVOTO	Aortic valve malformation	BAV - RL, RN or other
☐	LVOTO	Aortic valve malformation	Aortic Valve Malformations (nos)
☐	LVOTO	Mitral valve malformation	MVP
☐	LVOTO	Mitral valve malformation	MS
☐	LVOTO	Multiple obstruction	Shone Anomaly variant
☐	RVOTO	Pulm valve stenosis	PVS
☐	RVOTO	Pulm valve stenosis	PVS, nos
☐	RVOTO	Tricuspid atresia	Tricuspid atresia, IVS
☐	RVOTO	Tricuspid atresia	Tricuspid atresia + VSD
☐	RVOTO	Ebstein's	Ebstein
☐	RVOTO	Pulm atresia	PA-IVS
☐	RVOTO	Pulm atresia	PA, nos
☐	RVOTO	Pulm atresia	PA-VSD (not TOF anatomy)
☐	RVOTO	Pulm atresia	PA-VSD, nos
☐	RVOTO	Pulm atresia | Ebstein's	PA-IVS + Ebstein
☐	RVOTO	Tricuspid atresia | Pulm atresia	Tricuspid atresia + PA-IVS
☐	RVOTO	Anatomic Peripheral PS	Anatomic Peripheral PS
☐	Septal	VSD, pm	VSD pm
☐	Septal	VSD, musc	VSD musc
☐	Septal	VSD, os/nos	VSD nos
☐	Septal	VSD, os/nos	VSD os
☐	Septal	ASD2	ASD2
☐	Septal	ASD, os/nos	ASD, nos
☐	Septal	ASD, os/nos	ASD, os
☐	Septal	VSD pm, musc, os/nos, conov	VSDs multiple (pm, musc, or nos)

Table S1. *Cont.*

	Level Three	Level Two	Level One
**Cardiac Malformations (*Read 1—continued*)**			
☐	Septal	VSD pm, musc, os/nos, conov | ASD2, ASD nos/os	VSD (non-inlet) + ASD2/ASD nos
☐	Septal	PFO at age > 1 yr	PFO at age ≥ 1 yr.
☐	Septal	PDA at age > 1 yr	PDA at age ≥ 1 yr.
☐	Heterotaxy	Heterotaxy	Heterotaxy or S.I.totalis, simple CVM
☐	Heterotaxy	Heterotaxy	Heterotaxy or S.I.totalis, complex CVM
☐	Heterotaxy	Heterotaxy	Heterotaxy or S.I.totalis, no CVM
☐	Heterotaxy	Heterotaxy	Right Isomerism (Asplenia)
☐	Heterotaxy	Heterotaxy	Left Isomerism (Polyplenia)
☐	Heterotaxy	Heterotaxy	Dextrocardia + mirror image strap vessels + normal intracardiac anatomy
☐	Complex	SV or other complex	Multiple, complex heart anomaly
☐	Complex	SV or other complex	SV
☐	Complex	SV or other complex	SV, DILV, nos
☐	Complex	SV or other complex	SV, DILV, l-malposition
☐	Complex	SV or other complex	SV, DILV, d-malposition
☐	Complex	SV or other complex	SV, DIRV
☐	Complex	SV or other complex	SV, os
☐	Complex	SV or other complex	SV, nos
☐	Complex	L-TGA	L-TGA
☐	Complex	L-TGA	L-TGA + RVOTO (AS, COA)
☐	Complex	L-TGA	L-TGA + LVOTO (PS)
☐	Conotruncal, AVSD	d-TGA | AVSD	d-TGA-IVS + AVSD
☐	Conotruncal, AVSD	d-TGA | AVSD	d-TGA-VSD + AVSD
☐	Conotruncal, AVSD	Fallot | AVSD	TOF + AVSD
☐	Conotruncal, AVSD	Fallot | AVSD	PA-VSD (TOF anatomy) + AVSD
☐	APVR, AVSD	TAPVR | AVSD	TAVPR + AVSD
☐	APVR, AVSD	PAPVR | AVSD	PAVPR + AVSD
☐	Septal, LVOTO	VSD pm, musc, os/nos, conov | Coarctation	VSD (non-inlet) + COA
☐	Septal, LVOTO	VSD pm, musc, os/nos, conov | ASD2, ASD nos/os | AS	VSD (non-inlet) + ASD2/nos + AS
☐	Septal, LVOTO	VSD pm, musc, os/nos, conov | ASD2, ASD nos/os | Coarctation	VSD (non-inlet) + ASD2/nos + COA
☐	Septal, RVOTO	ASD2 | Pulm valve stenosis	ASD + PVS
☐	Septal, RVOTO	VSD pm, musc, os/nos, conov |Pulm valve stenosis	VSD (non-inlet) + PVS
☐	Septal, RVOTO	VSD pm, musc, os/nos, conov | ASD2, ASD nos/os | Pulm valve stenosis	VSD (non-inlet) + ASD2/nos + PVS

Table S1. *Cont.*

	Level Three	Level Two	Level One
**Cardiac Disease (*Read 1—continued*)** *If applicable, select only one primary diagnosis from the Cardiac Disease section. If the relevant diagnosis is not listed, choose "Other" and specify at the bottom of the chart.*			
☐	Aortopathy	Aortopathy	Aortic root dilation (z ≥ 2.5)
☐	Aortopathy	Aortopathy	Ascending aorta dilation (z ≥ 2.5)
☐	Aortopathy	Aortopathy	Aortic sinotubular junction dilation (z ≥ 2.5)
☐	Arteriopathy	Vascular Anomaly	Right Aortic Arch
☐	Arteriopathy	Vascular Anomaly	Double Aortic Arch
☐	Arteriopathy	Vascular Anomaly	Aberrant Subclavian Artery
☐	Arteriopathy	Vascular Anomaly	PA Anomaly (nos)
☐	Arteriopathy	Vascular Anomaly	Vascular Ring (nos)
☐	Arteriopathy	Vascular Anomaly	Aortopulmonary Window
☐	Arteriopathy, LVOTO	Arteriopathy	Supravalvar AS
☐	Coronary Anomaly	Coronary Anomaly	ALCAPA
☐	Coronary Anomaly	Coronary Anomaly	Coronary Anomaly (nos)
☐	Cardiomyopathy	Cardiomyopathy	HCM
☐	Cardiomyopathy	Cardiomyopathy	DCM
☐	Cardiomyopathy	Cardiomyopathy	RCM
☐	Cardiomyopathy	Cardiomyopathy	ARVC
☐	Cardiomyopathy	Cardiomyopathy	LVNC
☐	Cardiomyopathy	Left Ventricular Dysfunction	Left Ventricular Dysfunction
**Other (*Read 1—continued*)** *This section is to be used when a primary diagnosis is not listed in the Cardiac Malformations and/or Cardiac Disease sections. It may also be used to record any secondary diagnoses relative to selections in the Cardiac Malformations and/or Cardiac Disease sections.*			
	**Level Three**	**Level Two**	**Level One**
☐	* Other, specify: ________	** Other, specify: ________	*** Other, specify: ________
☐	* Other, specify: ________	** Other, specify: ________	*** Other, specify: ________
☐	* Other, specify: ________	** Other, specify: ________	*** Other, specify: ________

* Level 3: For "Other", indicate any of the listed category 3 diagnoses or specify an "other" unlisted classification; ** Level 2: For "Other", indicate any of the listed category 2 diagnoses or specify an "other" unlisted classification; *** Level 1: For "Other", indicate any of the listed category 1 diagnoses or specify an "other" unlisted classification.
